# Pseudolymphomatous Granuloma Annulare Rich in B Lymphocytes

**DOI:** 10.3390/dermatopathology13020019

**Published:** 2026-04-29

**Authors:** Angel Fernandez-Flores, José Luis Martínez-Amo

**Affiliations:** 1 Servicio de Anatomía Patológica, Hospital El Bierzo, Médicos sin Fronteras 7, 24411 Ponferrada, Spain; 2 Department of Cellular Pathology, Hospital de la Reina, 24400 Ponferrada, Spain; 3 Department of Dermatology, University Hospital Poniente, 04700 Almeria, Spain; jlmartinezamo@gmail.com

**Keywords:** granuloma annulare, pseudolymphomatous, cutaneous pseudolymphoma, differential diagnosis, dermatopathology

## Abstract

Granuloma annulare is a non-infectious granulomatous dermatosis with a probable pathogenic mechanism of delayed-type hypersensitivity, in which the dermal histiocytic granulomatous infiltrate is usually accompanied by a lesser component of lymphocytes. Although there are more common clinical and histopathological patterns of presentation, there are less well-known variants that may pose significant diagnostic challenges by mimicking other inflammatory cutaneous processes or even neoplastic conditions. One of the rarest forms of granuloma annulare is the pseudolymphomatous variant, in which the lymphocytic component is not only highly prominent but may, in some cases, partially or completely obscure the histiocytic component itself. This feature, together with the fact that the clinical presentation of this variant is often atypical—frequently lacking the characteristic annular morphology of conventional granuloma annulare—renders the diagnosis particularly challenging. From an immunohistochemical standpoint, the infiltrates described are predominantly composed of T cells, with only a sparse and scattered B-cell component. In this article, we present a case of granuloma annulare with a pseudolymphomatous B-cell component (PAX5^+^, CD79^+^) and minimal T-cell involvement, observed in a 4 mm skin nodule located on the shoulder of a 48-year-old male. This case therefore broadens the concept of pseudolymphomatous granuloma annulare to include infiltrates predominantly composed of B lymphocytes.

## Simple Summary

Pseudolymphomatous granuloma annulare was first described in 2012. Since then, the cases reported in the literature have consistently shown a predominantly T-cell lymphocytic component. We present a case with a predominance of B lymphocytes, which is particularly relevant in the differential diagnosis with primary cutaneous marginal-zone B-cell lymphoma accompanied by a granulomatous reaction.

## 1. Introduction

Granuloma annulare is a non-infectious granulomatous dermatosis of uncertain etiopathogenesis, although it is most likely mediated by immunological mechanisms, particularly type IV (delayed-type) hypersensitivity. Histopathologically, it is characterized by dermal infiltration by histiocytes, which often adopt a palisading arrangement around a central focus of collagen necrobiosis, accompanied by abundant mucin deposition.

From a clinical standpoint, granuloma annulare typically presents with asymptomatic or paucisymptomatic lesions, either solitary or multiple, manifesting as papules or plaques that are skin-colored, pink, or slightly erythematous, arranged in an annular or arcuate configuration with a relatively flat center. The disease follows a chronic course and, in many cases, resolves spontaneously.

Although this clinicopathological presentation is the most common, less frequent variants exist from both the clinical and histopathological perspectives. Clinically, these include localized, generalized or disseminated, subcutaneous, patch-type, and perforating forms, among others.

Histopathologically, the most common pattern is the palisading granulomatous pattern described above, with histiocytes surrounding a central area of necrobiosis. However, interstitial variants may also be observed, in which histiocytes are arranged as scattered cells within the dermis, intermingled with collagen bundles, and in which mucin deposition may be minimal or absent.

In both classic palisading granuloma annulare and its interstitial variant, it is not uncommon for the histiocytic infiltrate to be accompanied by a less prominent perivascular lymphocytic component. However, a variant of granuloma annulare exists in which the lymphocytic infiltrate is particularly prominent, at times masking the histiocytic component, thereby rendering the differential diagnosis with cutaneous lymphoma challenging. This variant is referred to as pseudolymphomatous granuloma annulare, of which only three cases to our knowledge have been reported in the literature [1,2,3]. 

Many nodular cutaneous lymphocytic infiltrates that raise concern for cutaneous lymphoma are predominantly composed of B lymphocytes, making cutaneous marginal-zone lymphoma and cutaneous follicular lymphoma the main differential diagnostic considerations. In this context, pseudolymphomatous granuloma annulare has not, until now, raised significant diagnostic difficulties in the literature once appropriate immunohistochemical studies were performed, as the accompanying lymphocytic infiltrate in previously reported cases has been composed predominantly of CD3-positive T lymphocytes (mixed CD4^+^/CD8^+^), with only scattered CD20^+^ B cells [2,3].

In contrast, in the present article, we describe an example of granuloma annulare with a unilesional papular clinical presentation, but with a histopathological pattern centered on a palisading granuloma with mucin-rich central necrobiosis, typical of granuloma annulare, and an accompanying predominantly B-cell peripheral lymphocytic infiltrate showing benign morphological, immunohistochemical, and molecular features. This finding broadens the conceptual and definitional spectrum of pseudolymphomatous granuloma annulare and supports the inclusion of this entity in the differential diagnosis of cutaneous B-cell pseudolymphomatous infiltrates. Likewise, it mandates careful distinction from cutaneous marginal-zone lymphoma with an associated granulomatous response, as well as with reactive inflammatory entities rich in B cells, such as, for example, lupus erythematosus-related lesions.

## 2. Case Presentation

**Figure 1:**
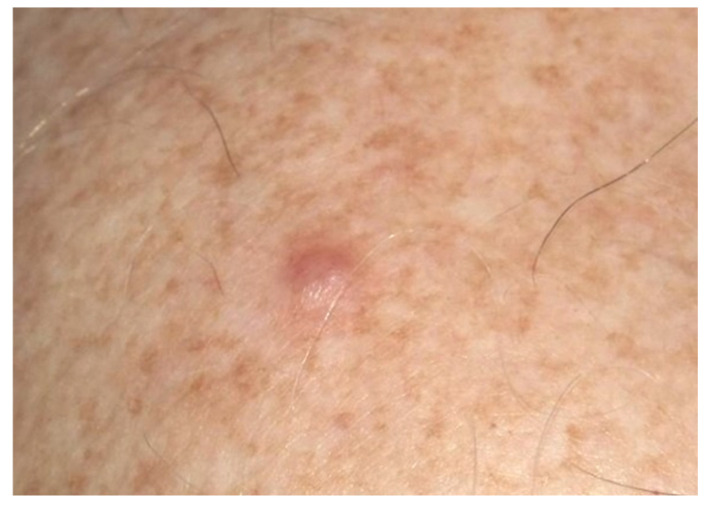
Clinical appearance of the lesion, corresponding to a slightly erythematous 4 mm nodule located on the right shoulder.

A 48-year-old male presented with a 4 mm nodular lesion on his right shoulder, which he had noticed in the last two months (Figure 1). The patient had no relevant personal or family medical history. The lesion was excised under the clinical suspicion of basal cell carcinoma. 

**Figure 2:**
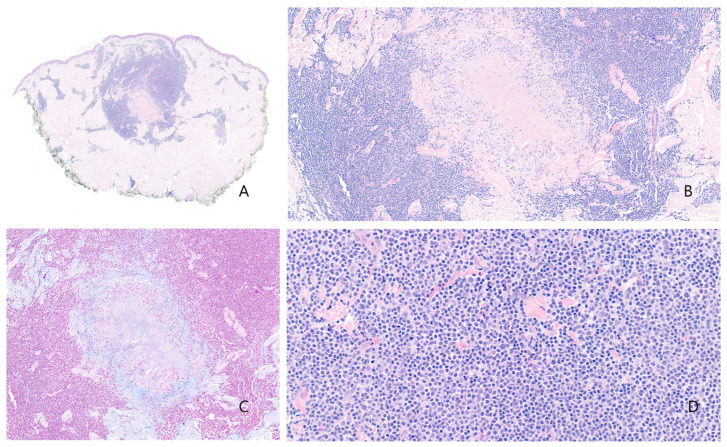
(**A**) Necrobiotic dermal granuloma with morphological features consistent with granuloma annulare. At this magnification, the density of the surrounding lymphoid infiltrate is clearly evident. (**B**) The necrobiotic centre of the granuloma is visible, along with the characteristic palisading arrangement of histiocytes typical of granuloma annulare. (**C**) Alcian blue histochemical staining reveals mucin deposition in the centre of the granuloma. (**D**) The inflammatory infiltrate consisted of lymphocytes, including centrocytes, centroblasts, immunoblasts, and occasional mature plasma cells. No eosinophils were observed.

Histopathological examination revealed a central necrobiotic granuloma, with collagen degeneration and mucin deposition, surrounded by palisading epithelioid histiocytes (Figure 2A–C). No caseating necrosis was observed. Special histochemical stains—including periodic acid–Schiff (PAS), Ziehl–Neelsen, Grocott, and Fite—did not reveal any microorganisms. The examination of the biopsy under polarized light revealed no birefringent particles or arthropod components.

**Figure 3:**
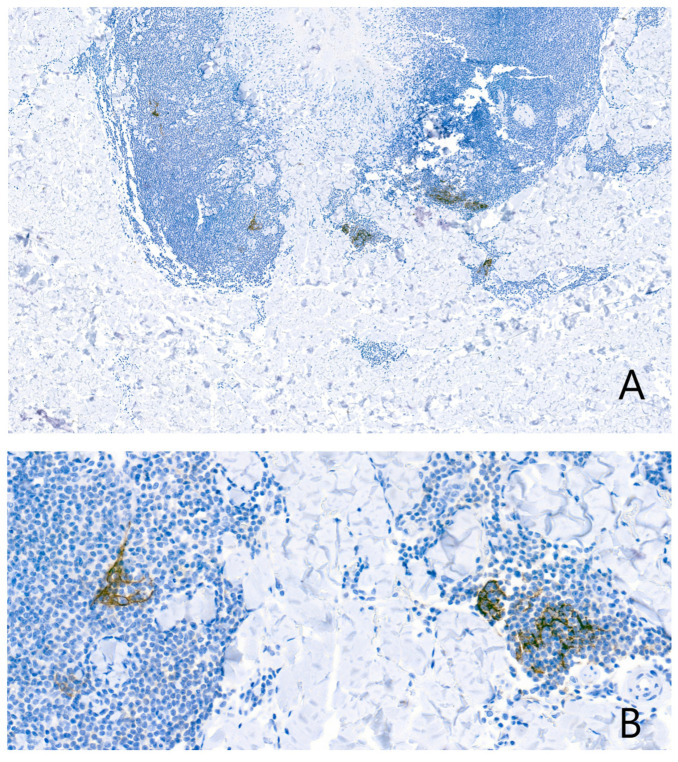
The CD21 study revealed three small residual germinal centres in the deepest portion of the infiltrate, represented by these networks of dendritic cells. (**A**) low magnification; (**B**) high magnification.

**Figure 4:**
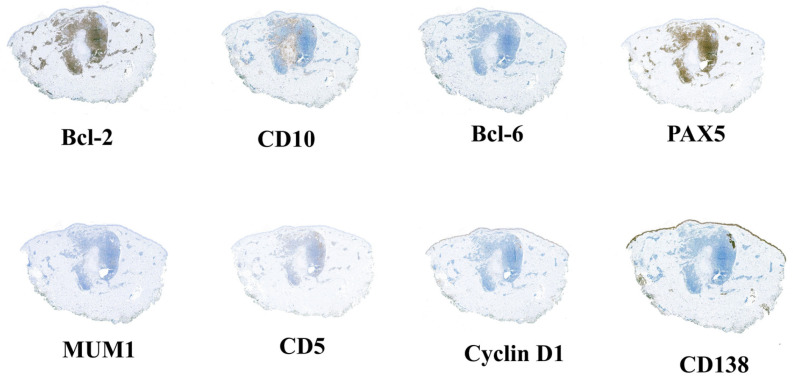
Immunophenotyping of the infiltrate showing B-cell lineage (positive for PAX5 and Bcl-2), with negativity for MUM1, CD5, Cyclin D1, CD10, Bcl6, and CD138.

**Figure 5:**
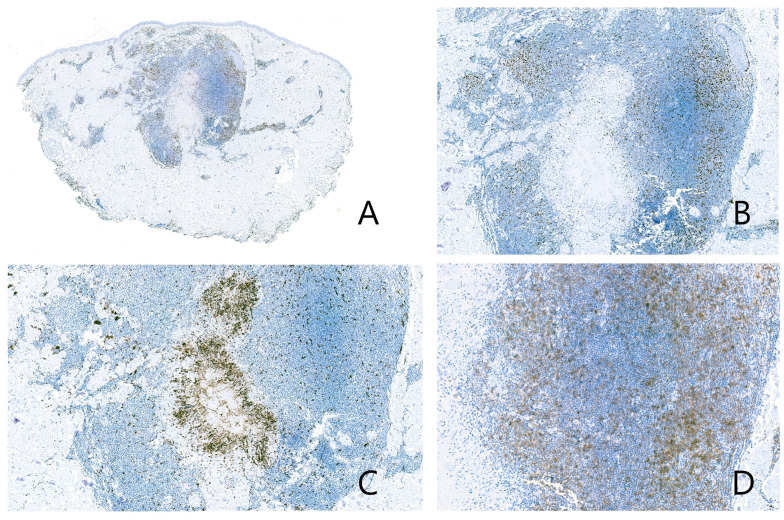
(**A**) CD3 immunostaining highlights a small population of T cells within the dense B-cell infiltrate. (**B**) The proliferation index (Ki-67) was low. (**C**) CD68 (PGM1) strongly stained the histiocytes of the granuloma, emphasising their palisading arrangement. (**D**) CD23 revealed a moderate number of scattered positive lymphoid cells within the infiltrate.

**Figure 6:**
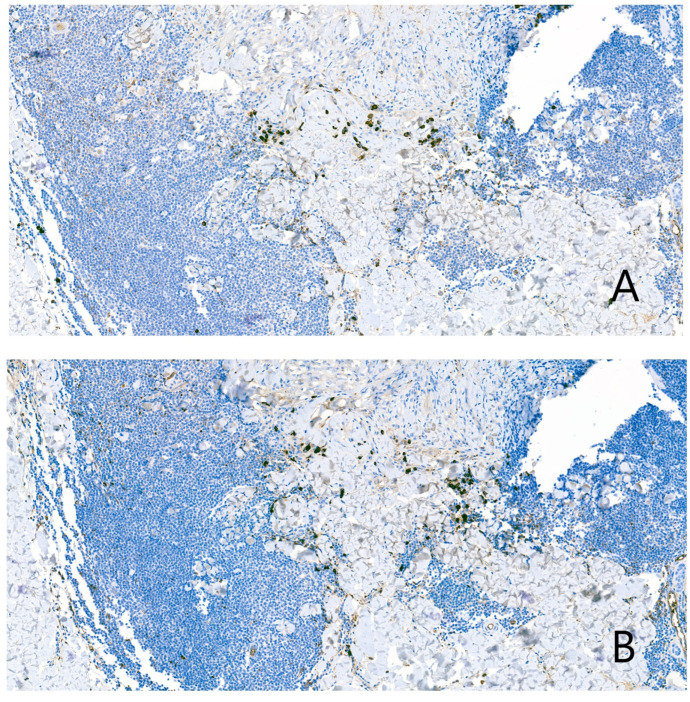
Immunostaining for kappa (**A**) and lambda (**B**) light chains demonstrated a polytypic staining pattern in the few plasma cells present within the infiltrate.

Surrounding the granuloma annulare was a lymphoid population composed of lymphocytes with morphology suggestive of centrocytes, centroblasts, and immunoblasts (Figure 2D), with a scant number of mature plasma cells. There were no well-formed lymphoid follicles or tingible body macrophages, although the CD21 study revealed three small residual aggregates of dendritic cells in the deepest portion of the infiltrate (Figure 3A,B). Eosinophils were not present in the infiltrate. The lymphoid cells were predominantly B cells (95% B cells; 5% T cells) (CD79^+^, PAX5^+^, CD20^−^, CD138^−^) expressing Bcl-2, but negative for CD10, Bcl-6, Cyclin D1, SOX11, CD5, and MUM1 (Figure 4). T cells (CD3^+^) were few and scattered (Figure 5A). The proliferation index (Ki-67) was low (10%) (Figure 5B). CD68 stained the histiocytes of the granuloma and also highlighted a component of small histiocytic cells scattered throughout the lymphoid infiltrate (Figure 5C). CD23 marked a moderate number of scattered cells within the infiltrate (Figure 5D). No aggregates of plasmacytoid dendritic cells were identified with CD123, which showed weak staining of granuloma histiocytes. Immunostaining for kappa and lambda light chains demonstrated a polyclonal staining pattern in the few plasma cells present (Figure 6). No CD1a-positive microorganisms were identified (Novocastra antibody).

A FISH analysis was performed using the Dako-Agilent IgH IQFISH Break-Apart probe to detect rearrangements at 14q32.33, targeting the immunoglobulin heavy-chain (IgH) locus. No rearrangements were identified.

A serological study for anti-*Borrelia burgdorferi* antibodies was performed on peripheral blood, showing IgM values of 0.17 index units (negative) and IgG values of 8.87 UA/mL (negative). All other laboratory parameters were within normal limits, except for a mild lymphocytosis (4.25 × 10^3^/µL; 49.6%) with a preserved total leukocyte count (8.57 × 10^3^ leukocytes/µL). No lymphadenopathy was detected on physical examination. At the time of reporting this case (three months after the patient’s initial evaluation), no recurrences or new lesions have been observed.

## 3. Discussion

The pathogenesis of granuloma annulare is not completely understood; however, it is now widely accepted that it represents an immune-mediated dermatosis, with a predominance of delayed-type (type IV) hypersensitivity mechanisms directed against an unknown antigen, and involving the coordinated participation of T lymphocytes, histiocytes, and proinflammatory cytokines, which induce dermal collagen damage with subsequent mucin deposition in many cases. Among the most frequently cited triggering factors in the literature are insect bites, trauma, infections, and vaccinations.

Regardless of the initiating trigger, activation of CD4-positive T lymphocytes—predominantly of the Th1 subset—is thought to occur, leading to the release of proinflammatory cytokines and recruitment of histiocytes. The release of interferon-gamma (IFN-γ), tumor necrosis factor-alpha (TNF-α), interleukin-2 (IL-2), and interleukin-12 (IL-12) results in macrophage activation, palisading arrangement, and the production of lysosomal enzymes and metalloproteinases, which in turn induce collagen degeneration (necrobiosis) and remodeling of the extracellular matrix. The mucin deposition frequently observed in granuloma annulare is most likely caused by cytokine-mediated fibroblast stimulation, with increased production of glycosaminoglycans (particularly hyaluronic acid) and decreased local degradation. This pathogenetic framework explains the non-infectious nature of the condition as well as its tendency toward spontaneous resolution. It also accounts for the fact that the accompanying lymphocytic component observed in granuloma annulare is predominantly T cell in nature (CD3-positive).

This CD3-positive lymphocytic component may at times be particularly prominent, occasionally obscuring the histiocytic component, thereby giving rise to what has been termed the pseudolymphomatous variant of granuloma annulare. In this setting, a histiocytic granulomatous reaction typical of granuloma annulare is accompanied by a reactive infiltrate, predominantly composed of mixed CD4^+^ and CD8^+^ T cells [3]. The infiltrate is typically distributed around superficial and deep dermal vessels, without lymphocytic atypia, and coexists with an interstitial or necrobiotic pattern of granuloma annulare [3]. Clinical suspicion of granuloma annulare is raised in only approximately 30% of cases, and the lesions are most often localized [3]. In cases in which granuloma annulare is not clinically suspected, the presentation usually consists of papules or plaques lacking a clearly annular configuration. Consequently, these lesions lack distinctive specific features and may mimic a wide spectrum of inflammatory and lymphoproliferative dermatoses, making clinicopathologic correlation essential for an accurate diagnosis.

From a histopathologic standpoint, lymphocytic infiltrates may often obscure the typical features of granuloma annulare [3], rendering the diagnosis more challenging, as this variant most commonly exhibits an incomplete interstitial pattern, while the classic necrobiotic presentation is observed in only a minority of cases.

The literature emphasizes that one of the key features in the differential diagnosis with B-cell lymphoma is that the lymphoid infiltrate is predominantly composed of T lymphocytes, with a smaller accompanying population of CD68-positive and CD163-positive histiocytes [3]. However, our case proved to be atypical in that it represents a pseudolymphomatous granuloma annulare with an exuberant B-cell lymphocytic component and only a minimal T-cell component. This feature requires a careful differential diagnosis with cutaneous B-cell lymphoma, particularly marginal-zone lymphoma and follicular lymphoma. In this context, it is crucial to identify the necrobiosis-centered granulomatous response, which in our case constituted the core of the lymphoid proliferation, and to demonstrate collagen necrobiosis with associated mucin deposition, in contrast to the expansile and destructive growth pattern typically observed in lymphomatous infiltrates, which are not subordinated to a central granulomatous response. The presence of CD21-positive remnants is characteristic of residual dendritic cell networks within a secondary inflammatory reaction. No lymphoid tumor nodules were identified. Marginal-zone lymphoma usually presents with prominent germinal center follicles and often shows differentiation toward abundant mature plasma cells that typically display monotypic expression of immunoglobulin light chains, none of which was observed in this case. It should be recalled that a variant of marginal-zone lymphoma exists in which an associated granulomatous response is present, most likely resulting from chronic antigenic stimulation induced by the lymphoma itself. However, in this variant, the epithelioid granulomas are usually located at the periphery of the lymphoma and display a sarcoidal, interstitial, or poorly defined morphology, without associated necrobiosis or mucin deposition.

In our view, the present lesion is best interpreted as pseudolymphomatous granuloma annulare with an unusually B-cell-predominant lymphoid component, rather than as primary cutaneous B-cell lymphoma with a secondary granulomatous reaction. This interpretation is based on the fact that the lesion is architecturally centered on a well-formed necrobiotic and mucin-rich palisading granuloma, which represents the classic histopathologic hallmark of granuloma annulare. In contrast, previously reported cases of pseudolymphomatous granuloma annulare have likewise emphasized that the decisive clue is the recognition of bona fide granuloma annulare masked by a dense lymphoid infiltrate, even when the clinical presentation is not suggestive of granuloma annulare. In addition, primary cutaneous marginal-zone lymphoma usually shows a nodular or diffuse lymphoid proliferation with conspicuous reactive germinal centers and often plasmacytic differentiation, frequently accompanied by light-chain restriction; these features were not present in our case. Likewise, primary cutaneous follicle center lymphoma is typically supported by a follicle center phenotype, especially BCL6 and/or CD10 expression in the appropriate morphologic setting, which was also absent here. Therefore, we believe that the central diagnostic argument is not merely the coexistence of granulomatous and lymphoid components, but the fact that the lymphoid infiltrate appears subordinated to a typical granuloma annulare-like necrobiotic–mucinous core rather than forming an autonomous expansile B-cell proliferation. Nevertheless, because the clinical follow-up is limited, this interpretation should be regarded as strongly favored rather than absolutely proven.

From a cytological standpoint, the lymphoid response observed in our case was polymorphous, without nuclear atypia and without predominance of any single lymphoid subpopulation. In particular, centro-germinal markers characteristic of follicular lymphoma, such as BCL6 and CD10, were negative, and immunoglobulin light-chain expression was polytypic, in contrast to what is typically observed in cutaneous marginal-zone lymphoma. Although our case showed CD23 expression in scattered cells, these cells did not co-express CD5, which could otherwise suggest a small lymphocytic lymphoma. We also employed typical markers of mantle cell lymphoma, such as cyclin D1 and SOX11, both of which were negative.

An alternative possibility is that the infiltrate in this case represents a B-cell pseudolymphoma secondary to an arthropod bite. Supporting this hypothesis is the clinical presentation as a solitary nodule, rather than the more typical distribution of granuloma annulare. However, this possibility is questioned by the absence of eosinophils and the presence of a necrobiotic granuloma with central mucin deposition. Although persistent insect bite reactions have historically been referred to as “dermal eosinophilic granulomas” [4], such infiltrates more closely resemble what would now be considered pseudolymphomas with histiocytes, rather than well-formed granulomas, particularly of the necrobiotic type. Nonetheless, it is currently recognised that arthropod bites are one of the hypothetical aetiological triggers of granuloma annulare, and some cases have been linked to prior insect bites [5].

Another diagnostic possibility to consider is *Borrelia* infection (which was ruled out in this case). In fact, a study analysing 157 biopsies of granuloma annulare detected Borrelia in 127 cases (80.9%) [6], with the highest frequency observed in cases of localized GA (102 out of 120; 85%) [6]. The authors of that study proposed that Borrelia may play a role in the pathogenesis of granuloma annulare, particularly in endemic regions, which does not apply to our setting. Additionally, in large series on Borrelia burgdorferi-associated lymphocytoma cutis, it has been demonstrated that prominent germinal centers with evidence of tingible body macrophages are a consistent feature [7].

Moreover, Borrelia infection is well known to be associated with B-cell pseudolymphomas. In a study of 106 patients with 108 biopsies of Borrelia-associated pseudolymphoma, the predominant pattern was that of a dense diffuse infiltrate, although no cases were associated with granuloma annulare [7]. Notably, in five cases, the infiltrate simulated diffuse large B-cell lymphoma, and in two cases, clonal IgH rearrangement was demonstrated by polymerase chain reaction (PCR) [7]. In contrast to diffuse large B-cell lymphomas, such expanded germinal centers show preservation of dendritic cells and usually retain germinal center markers BCL-6 and CD10, neither of which was present in our case. We also found no expression of the activation marker MUM1, which is often demonstrated in many diffuse large B-cell lymphomas of post-germinal center phenotype.

In the differential diagnosis of pseudolymphomatous granuloma annulare, lupus erythematosus in its various forms—particularly tumid lupus—should be considered, as well as mycosis fungoides, especially its interstitial variant [8], and granulomatous drug reactions. The clinical information provided plays a crucial role in establishing the diagnosis of lupus erythematosus or a drug-induced secondary reaction. In interstitial mycosis fungoides, histiocytes are never more numerous than lymphocytes. In contrast, in granulomatous mycosis fungoides, the granulomatous component may often obscure the underlying lymphoid neoplastic component; however, such granulomas are not necrobiotic. In all of these entities, the lymphocytic infiltrate is predominantly composed of T cells, a scenario that was not observed in our case. We specifically investigated plasmacytoid dendritic cell positivity using CD123 in order to exclude a lupus erythematosus-related lesion. In this regard, it should be noted that histiocytes may occasionally exhibit CD123 positivity, which can generate diagnostic confusion in lesions of a granulomatous nature.

Additionally, among dermal infiltrates characterized by B-cell-rich lymphoid responses accompanied by granulomas, leishmaniasis should be specifically highlighted. In such cases, plasma cells commonly accompany the lymphoid infiltrate and are often numerous [9]. Furthermore, the granulomas observed typically display a characteristic morphology described as “messy” [10,11]. In our case, no amastigotes were identified on multiple histologic sections, and CD1a immunohistochemistry failed to demonstrate microorganisms. Likewise, no “messy”-type granulomas were observed.

A limitation of the present report is the relatively short clinical follow-up, which currently extends to only three months. Although the morphologic, immunophenotypic, and ancillary findings favor a reactive/pseudolymphomatous process over an indolent primary cutaneous B-cell lymphoma, this interval is too short to exclude such a lymphoma with complete confidence. Accordingly, our interpretation should be regarded as strongly favored on clinicopathologic grounds, but deserving of continued clinical follow-up.

## 4. Conclusions

This case combines the typical histopathological features of granuloma annulare with those of a B-cell pseudolymphoma and thereby broadens the concept of pseudolymphomatous granuloma annulare to include cases with predominantly B-cell lymphoid infiltrates. This case further emphasizes the variability in the clinical presentation of pseudolymphomatous granuloma annulare, which in many instances may lack the classic clinical features of granuloma annulare, yet may still be suggested by histopathologic findings of granulomas with a mucin-rich necrobiotic center. 

From a practical diagnostic standpoint, this case suggests that B-cell-predominant pseudolymphomatous granuloma annulare may be considered when the following features coexist: (1) a central, well-formed palisading necrobiotic granuloma with conspicuous mucin deposition, histopathologically typical of granuloma annulare; (2) a dense surrounding lymphoid infiltrate that is architecturally subordinated to the granulomatous process rather than forming an autonomous expansile lymphoid proliferation; (3) a polymorphous B-cell-rich infiltrate lacking significant cytologic atypia; (4) absence of well-developed neoplastic follicles or other architectural features supporting primary cutaneous B-cell lymphoma; (5) absence of monotypic plasma-cell differentiation or light-chain restriction; (6) lack of a follicle center phenotype, with no supportive expression of markers such as CD10 or BCL6 in the lesional B cells; and (7) ancillary studies that fail to support lymphoma, interpreted in the context of clinicopathologic correlation. These features should not be regarded as definitive criteria on the basis of a single case, but rather as practical diagnostic clues that may help distinguish this lesion from primary cutaneous marginal-zone lymphoma with granulomatous reaction and from other reactive B-cell-rich cutaneous pseudolymphomatous infiltrates.

## Data Availability

The data presented in this study are available on request from the corresponding author.
